# Psychological interventions for individuals with Ehlers-Danlos syndrome and hypermobility spectrum disorder: a scoping review

**DOI:** 10.1186/s13023-023-02799-y

**Published:** 2023-08-31

**Authors:** Jessica Z. Song, Dorothy Luong, Estée C. H. Feldman, Susan Tran, Laure Perrier, Kathleen Eubanks, Mark Bayley, Monika Kastner, Maxwell Slepian, Sarah E. P. Munce

**Affiliations:** 1 KITE Research Institute, Toronto Rehabilitation Institute - University Health Network, Toronto, ON Canada; 2 Behavioral Medicine and Clinical Psychology, Cincinnati Children’s Hospital Medical Center, Cincinnati, OH USA; 3 Department of Psychology, DePaul University, Chicago, IL USA; 4 Dalla Lana School of Public Health, Institute of Health Policy, Management and Evaluation, University of Toronto, Toronto, ON Canada; 5 Ehlers-Danlos Syndrome Canada, Toronto, ON Canada; 6 North York General Hospital, Toronto, ON Canada; 7 GoodHope Ehlers-Danlos Syndrome Clinic, Toronto General Hospital, University Health Network, Toronto, ON Canada; 8 Institute of Health Policy, Management and Evaluation, University of Toronto, Toronto, ON Canada; 9 Department of Anesthesia and Pain Management, Toronto General Hospital, University Health Network, Toronto, ON Canada; 10 Krembil Research Institute, University Health Network, Toronto, ON Canada; 11 Department of Anesthesiology and Pain Medicine, University of Toronto, Toronto, ON Canada; 12 Department of Occupational Science and Occupational Therapy, University of Toronto, Toronto, ON Canada

**Keywords:** Ehlers-Danlos syndrome, Joint hypermobility, Joint hypermobility syndrome, Psychological interventions, Cognitive behavioural therapy, Hypermobility spectrum disorder, Chronic pain

## Abstract

**Purpose:**

To identify the nature and extent of the evidence on psychological interventions among individuals with Ehlers-Danlos Syndrome (EDS) and Hypermobility Spectrum Disorder (HSD).

**Materials and methods:**

Eligible studies reported on psychological interventions for individuals of all ages with EDS and/or HSD. All studies published in English were included, with no restrictions to publication year or status. MEDLINE, CINAHL, EMBASE, and PsycINFO were searched. Two reviewers independently screened studies and abstracted data.

**Results:**

This scoping review included 10 studies reporting on EDS, HSD, or both. Only cohort studies and case studies were identified. Four studies investigated Cognitive Behavioural Therapy (CBT), one investigated Dialectical Behavioural Therapy (DBT), two investigated psychoeducation, two investigated Intensive Interdisciplinary Pain Treatment (IIPT), and one investigated Acceptance Commitment Therapy (ACT). Interventions targeted pain management, self-destructive behaviours, and related psychological issues (e.g., depression/anxiety). Sample sizes were small (n < 50) for most studies and interventions were generally poorly described.

**Conclusions:**

There is a critical need for high-quality research surrounding psychological interventions for individuals with EDS/HSD. Psychological interventions for these individuals are understudied and existing studies lack validity. Researchers should investigate psychological interventions for individuals with all types of EDS/HSD with high-quality studies to validate findings from the existing studies.

**Supplementary Information:**

The online version contains supplementary material available at 10.1186/s13023-023-02799-y.

## Introduction

### Background

Ehlers-Danlos Syndrome (EDS) comprises a group of rare hereditary connective tissue disorders characterized by several shared clinical features such as joint hypermobility and hyper-extensible skin [1]. EDS is estimated to affect between 1 in 2500 and 1 in 5000 people globally [2]. Many individuals with EDS have frailty of soft tissue and blood vessels among other conditions which may contribute to chronic and severe disabilities, greatly affecting their quality of life and mortality [1]. While some individuals are asymptomatic for their entire lives, many others experience multi-systemic and multi-factorial complications, including neurological, gastrointestinal, and cardiovascular complications, breathing difficulties, organ rupture, joint dislocation, and chronic pain [2–5]. Currently, thirteen subtypes have been officially recognized, all of which are caused by different gene variants and have different manifestations [2, 6]. The six predominant types of EDS are classical, hypermobile, vascular, kyphoscoliotic, arthrochalasis, and dermatosparaxis. Classical, hypermobile and vascular EDS are the most prevalent, accounting for 79% of the EDS population [6], while hypermobile EDS (formerly EDS type III) is the most common subtype overall [7].

Hypermobility Spectrum Disorder (HSD), formerly known as Joint Hypermobility Syndrome (JHS), is diagnosed in individuals with generalized joint hypermobility, fatigue, and chronic pain [8]. HSD/JHS is occasionally classified as a milder variant of hypermobile EDS by health authorities because the two conditions share many clinical features, although this classification is not widely used [9]. The condition is prevalent in at least 3% of the general population and it is diagnosed only after other conditions, including any of the EDS subtypes, have been excluded [9]. Patients with HSD are often predisposed to soft tissue injury as well as slow and incomplete healing [10]. Complications associated with HSD may inlude central and peripheral nervous system abnormalities; for example, many experience increased pain, resistance to local anesthetics, and gastrointestinal complications [10].

There is no known cure for EDS or HSD, and they are often difficult to diagnose since clinical presentation may vary greatly between patients and there is a lack of specific genetic tests [11]. Many symptoms of EDS and HSD are also shared with other diagnoses, such as chronic fatigue syndrome, Marfan syndrome, and osteogenesis imperfecta [12, 13]. These difficulties often lead to a greater time to diagnosis compared to other diagnoses with similar symptom profiles (e.g., Marfan syndrome), which may be easily identified clinically and genetically [14, 15]. In a qualitative study of adults with EDS, it was revealed that many had shown symptoms as children that were overlooked and misdiagnosed by their families and healthcare professionals [16]. This makes EDS and HSD of concern from a lifespan perspective, as lifelong health complaints are rarely investigated but are important to address throughout a person’s development. In the qualitative study, those who were diagnosed as adults experienced unbearable pain and fatigue as their unaddressed symptoms worsened during their development into adulthood [16].

### Psychiatric burden of EDS and HSD

In addition to the physiological impairments mentioned above, psychiatric disorders and psychosocial impairment are also common among individuals with EDS and HSD. Specifically, EDS and HSD have been associated with increased risk of conditions such as depression and anxiety as well as interpersonal issues when compared to the general population [17–21]. In a cohort study of 106 patients with hypermobility-type EDS, Hershenfeld et al. [22] determined that psychiatric disorders were found in 42.5% of the cohort, with 22.7% of patients affected with 2 or more psychiatric diagnoses. Anxiety and depression were the most reported, with frequencies of 23.6% and 25.5%, respectively. This is notably higher compared to 17.6% of psychiatric disorders in the general population [23]. Moreover, the presence of any pain symptom was associated with nearly 10 times greater odds of having a psychiatric disorder.

The exact reasons for the high rates of psychiatric disorders are unclear, but it is likely that the aforementioned physical symptoms (e.g., chronic pain and other medically unexplained symptoms) play a major role [20]. Notably, the high prevalence of chronic pain in the EDS population is one of the many contributing factors that are hypothesized to be associated with high rates of psychosocial impairment [19]. For example, many patients are misdiagnosed for years or accused of malingering, leading to mistrust of the medical/health care system and self-doubt [19]. Furthermore, previous research has demonstrated that individuals with joint hypermobility have an altered sense of body awareness, which may play a role in emotional response and has been shown to mediate the relationship between joint hypermobility and anxiety [21]. For example, Mallorquí-Bagué et al. [24] found that hypermobile individuals experienced significantly higher state anxiety compared to those who were not hypermobile and that this relationship was mediated by interoceptive sensitivity (i.e., enhanced body awareness).

### Importance of psychological interventions

EDS may be managed with a variety of treatment options including physical therapy, nonsteroidal anti-inflammatory drugs, acetaminophen, opioid medications, and surgery, as well as non-traditional or complementary modalities including acupuncture and massage [25]. However, previous studies by Rombaut and colleagues [26] and Grahame [27] highlighted limitations of common pharmacological and physical interventions, noting that analgesics, non-steroidal anti-inflammatory drugs, psychotropics, and physiotherapy are often ineffective to address pain among individuals with EDS. In addition, individuals with EDS experience increased side effects of pain medications [28]; orthopedic procedures are indicated for pain relief in only selected patients [29]. Thus, it has been suggested that psychological approaches [30, 31], particularly to address chronic pain observed among individuals with EDS, may be helpful for recovery and rehabilitation. In a pilot study of 12 women with EDS, Bathen and colleagues [32] investigated a multidisciplinary rehabilitation program combining physical and cognitive behavioural therapy (CBT). Decreases in self-perceived pain were reported as well as increased participation in daily life. Such findings parallel those seen in patients with chronic pain [33], suggesting that such interventions may be readily adapted to meet the needs of patients with EDS and HSD.

### Gaps in research on psychological interventions

Despite these promising outcomes, it has been suggested that more research is needed on relevant psychosocial strategies for individuals with EDS [31]. Rombaut and colleagues [26] also emphasized the need for evidence-based recommendations for the optimal management of EDS, which includes psychological follow-up. Similarly, there is a lack of high-quality clinical guidelines for managing the psychosocial complexities associated with pain in EDS, limiting evidence-based practice [34]. Furthermore, patient partners have been previously recognized as important contributors to developing (psychosocial) treatment options in EDS, but their expertise has not been leveraged to date [34]. Overall, our study aims to examine the nature and extent of the current evidence on psychological interventions for individuals with EDS and HSD, identifying any gaps in research and providing suggestions for future research areas.

## Methods

### Design and search strategy and information sources

This review used the Joanna Briggs Institute methodological framework for the current scoping review with reference to Arksey and O’Malley’s framework as well [35, 36]. These frameworks outline six main stages involved in a scoping review: (1) identifying the research question; (2) identifying relevant studies; (3) selecting studies; (4) charting the data; (5) collating, summarising and reporting the results; and (6) consulting with relevant stakeholders. Scoping reviews are conducted to determine the nature of existing evidence and to analyze and identify knowledge gaps, rather than to provide a detailed appraisal and assessment of the quality of the literature [35]. Thus, a critical appraisal of the included studies is not required. The last stage of our project will be achieved via ongoing consultation with our integrated knowledge translation (iKT) Advisory Group, consisting of individuals with EDS and HSD. The overall project uses a collaborative iKT approach that engages all relevant knowledge users and stakeholders (i.e., researchers, clinicians, and patient partners) in the full spectrum of research activities such as setting the objectives and methods and implementation of the research [37]. Our patient partners also contributed to informing our search strategy development and interpretation of results. The PRISMA-ScR checklist guided the reporting of this review [38].

### Eligibility criteria

Studies involving the use of psychological interventions for individuals with EDS and/or HSD were included. We defined psychological interventions as those that aim to enhance coping strategies (e.g., for pain and resiliency) and address stress or low mood, either alone or in combination. This definition was previously used for a Cochrane systematic review and meta-analysis on psychological interventions for coronary heart disease [39]. Examples of psychological interventions include cognitive behavioural therapy (CBT), motivational interviewing, acceptance and commitment therapy (ACT), and mindfulness. We only included studies where the psychological interventions were delivered by health care professionals who had been trained in their delivery (including both individuals who hold a clinical degree and those whose training was described within the study). Studies with participants of all ages were included. Our scoping review included experimental studies (randomized controlled trials (RCTs), cluster RCTs, quasi-randomized trials, and controlled clinical trials), quasi-experimental studies (including interrupted time series and controlled before and after studies), observational studies, and qualitative studies. We included studies regardless of publication status, including the grey literature. Our search strategy included English-language studies only and had no restrictions with respect to publication year.

### Search strategy and information sources

Literature search strategies using subject headings and text words related to psychological interventions for individuals with EDS and/or HSD were developed from two systematic reviews [39, 40], as well as the search strategy used by our research team on psychological interventions for adults with cerebral palsy, childhood-onset acquired brain injury, and spina bifida [41]. A sample search strategy from CINAHL can be found in Additional file 1. A librarian with extensive experience in conducting systematic and scoping reviews conducted all of the literature searches in consultation with the research team. Experts in the field of EDS and HSD, including members of our research team, were consulted to ensure that all relevant data was obtained. A comprehensive literature search was run in Ovid MEDLINE, Ovid MEDLINE Daily, Ovid MEDLINE In-Process and Other Non-Indexed Citations, Ovid MEDLINE Epub Ahead of Print, OVID EMBASE, OVID PsycINFO, and EBSCOhost CINAHL on March 28, 2021. Duplicates were removed using Zotero's duplicate identification strategy as well as manually. Appropriate wildcards were used in the search to account for plurals and variations in spelling. A grey literature search was performed by searching targeted websites (i.e., Ehlers-Danlos Society, Ehlers-Danlos Support UK, Marfan Foundation) as recommended by members of our research team. The team developed inclusion and exclusion criteria screening questions and forms for the title and abstract screening (level 1 screening), full-text screening (level 2 screening), and abstraction.

### Selection of articles and data abstraction

To increase the reliability of screening among reviewers, a pilot test of a pre-defined screening form based on the eligibility criteria was performed on a random 1% sample, and reviewers reported 90% agreement or higher before moving on to full screening. The inclusion and exclusion criteria were discussed and clarified to promote the consistent application of the selection criteria if necessary. All title and abstract screening (i.e., level 1 screening) identified by the literature search were performed independently, in duplicate. The full text of the potentially relevant articles was acquired and screened to determine final inclusion (i.e., level 2 screening). Level 2 screening was performed independently and in duplicate. Discrepancies were resolved by discussion or by a third reviewer, if necessary. Studies excluded during the screening phase were documented along with an explanation for exclusion.

Data abstracted from the publications included study characteristics (e.g., author names, year of publication, country of study conduct, study design, sample size, setting characteristics), participant characteristics (e.g., EDS and/or HSD, age, sex, race, etc.), psychological intervention characteristics, and outcome results. We used the Template for Intervention Description and Replication (TIDieR) checklist and guide [42] to inform the data abstraction on the psychological interventions. This guide includes how, when, where, and how much the intervention was delivered, as well as whether any modifications or tailoring were made/are recommended [43]. The data abstraction form was pilot-tested and modified accordingly. Data abstraction was performed independently and in duplicate. Discrepancies were resolved by discussion or by a third reviewer, if necessary. The data from this scoping review were summarised quantitatively using numerical counts and as well as qualitatively using content analysis. Data are organized in the tables by type of psychological intervention.

## Results

**Fig. 1:**
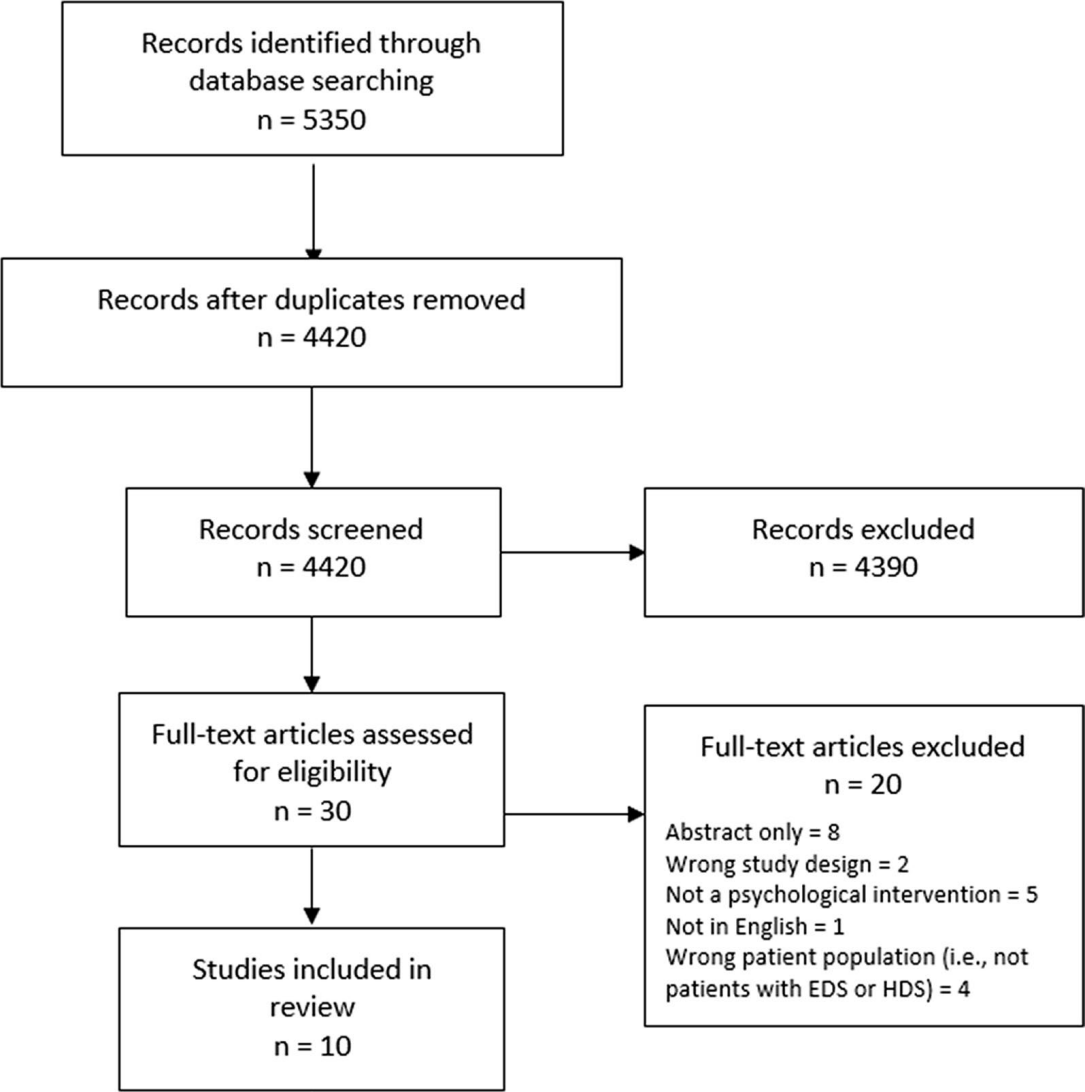
Articles yielded from the literature search, title and abstract screening, and full-text screening. Diagram adopted from PRISMA

MEDLINE, EMBASE, PsychINFO, and CINAHL retrieved 727, 4164, 128, and 331 studies, respectively. Our grey literature search found two unpublished reports from Ehlers-Danlos Support UK and one unpublished report from Ehlers-Danlos Society; the authors were contacted but we were unable to obtain the full reports. A flowchart of the results of the study selection process is provided in Fig. 1. The initial search yielded a total of 5350 articles; after duplicates were removed, 4420 articles underwent initial (level 1) screening. Subsequently, 30 articles were eligible for full-text screening, of which 20 were excluded, leaving 10 studies for data abstraction.

### Study characteristics

Table 1 highlights the study characteristics of the ten articles included in this review. The studies were published between 2011 and 2021. There were four case reports [44–47], five cohort studies [32, 48–51], and one study that featured two case reports and a literature review [52]. Three were conducted in the United States [47, 50, 51], two in the United Kingdom [46, 49], two in France [45, 48], one in Australia [44], one in Norway [32], and one in Canada [52].

**Table 1:** Description of included studies and study participants

Study	Study design	Conditions of participants	Country	N	Age range (years)	Sex/gender	Race/ethnicity	Education	Inclusion and exclusion criteria for participants
*Cognitive behavioural therapy (CBT)*									
Bathen et al. [32]	Cohort study (pre-test post-test)	Ehlers-Danlos Syndrome hypermobility type (EDS-HT) and Joint Hypermobility Syndrome (JHS)	Norway	12	20–51	12 female (100%)	Not described	13 years (n = 9) University/high school 1–3 years (n = 1) University/higher education 4 + years (n = 1) Missing (n = 1)	Inclusion criteria not explicitly stated; participants were all diagnosed with EDS-HT/JHS according to Villefranche criteria and had generalized hypermobility with Beighton score of 4/9 or more 4/9 or more combined with hypermobility in other joints. All participants had stretchiness of skin of 2.5 cm or more, or soft, velvety skin. All participants had chronic pain
Branson et al. [44]	Case report	EDS-HT and JHS	Australia	1	14	1 female (100%)	Unclear; born to Irish parents	First year of high school	Not described
Rahman et al. [49]	Cohort study	JHS	United Kingdom	130 attended the program, 87 attended 1 month follow-up, 65 attended 5-month follow-up	Range not provided; mean = 35	96% female	Not described	Not described	Participants must have JHS and presence of pain for over 3 months. Participants were excluded for the following: malignancy, inflammatory arthritis, significant suicidal ideation, using alcohol and/or illicit drugs to an extent that impaired cognitive function and concentration daily and inability to mobilise with-out a wheelchair
Zhou et al. [52]	Two case reports and a literature review (note that only case reports were extracted for data)	EDS (unspecified type), EDS-HT	Canada	2 (separate case reports)	Case 1: 41 Case 2: 23	2 female (100%)	Case 1: not described Case 2: Caucasian	Not described	Clinic patients; inclusion criteria not described
*Dialectical behavioural therapy (DBT)*									
Henry et al. [46]	Case report	Severe Emotional Unstable Personality Disorder (EUPD) with comorbid EDS (unspecified type) and Functional Neurological Disorder	United Kingdom	1	25	1 female (100%)	Caucasian	Not described	Clinic patient; inclusion criteria not described
*Psychoeducation*									
Chaleat-Valayer et al. [48]	Cohort study (prospective observational study)	EDS-HT	France	19 patients, 9 relatives, 28 total	39.2 (SD 15.2) for patients, 44.1 (SD 16.2) for relatives	Patients: 89% female Relatives: 22% female	Not described	Not described	Participants included all patients and family members who participated in one of 3 PrEduSED programs between 2014 and 2015 who gave consent to participation. The program was designed for patients with hEDS over 18 years old. Patients were diagnosed by one reference center and/or one geneticist, and relatives were not personally affected by the disease
Cravero et al. [45]	Case report	EDS (classic type) and Cornelia de Lange syndrome	France	1	21	1 male (100%)	Not described	Not described	Clinic patient; inclusion criteria not described
*Intensive interdisciplinary pain treatment (IIPT)*									
Randall et al. [50]	Cohort study (retrospective)	EDS, Complex Regional Pain Syndrome, diffuse body pain, juvenile fibromyalgia, back pain, idiopathic juvenile arthritis, abdominal pain, headache, other neuropathic pain disorder, and conversion (functional neurologic) disorder with pain symptoms	United States	217 attended the program, 95 returned questionnaires for the current study (5-year follow-up)	Range not provided; mean = 20 (SD = 2.5) at follow-up, 14.5 (SD = 2.5) at admission)	88% female	98% white	Attending college full time (n = 40) Attending college part-time (n = 5) Still completing secondary school (n = 32)	Participants must have at least 3 months’ duration of pain at admission, significant disruption in daily functioning due to pain, stable psychiatric status (i.e., no active suicidality or current need for inpatient level of psychiatric care), failure to make adequate functional progress in conventional outpatient physical and cognitive behavioural therapies
Revivo et al. [51]	Cohort study (retrospective)	EDS-HT, JHS	United States	30	9–18	27 female (90%)	Not described	Not described	Participants were 18 years of age and younger with joint hypermobility and were treated in the intensive interdisciplinary pain management program
*Acceptance and commitment therapy (ACT)*									
Knowlton et al. [47]	Case report	EDS (vascular type) and Postural Orthostatic Tachycardia Syndrome (POTS)	United States	1	Unclear; early 30 s	1 female (100%)	Caucasian	Not described	Clinic patient; inclusion criteria not described

JHS would be presently known as HSD

### Participant characteristics

There were 258 total participants in the ten studies included in our review. Sample sizes ranged from 1 (case report) to 95 (cohort study), with participants ranging in age from 9 to 51 years (note that some studies only provided a mean age, rather than an age range). Most studies (80%) had a small sample size (n < 50), which may be partly attributed to the study types (i.e., many were case studies). Nine of the ten studies included a higher proportion of female participants (90–100%) compared to males, with only one case study featuring a male patient. The ten studies included participants with EDS (hypermobile [32, 44, 48, 51, 52], classic [45], vascular [47], or unspecified [46, 50, 52] subtype) and/or JHS [32, 44, 49, 51]. JHS would be presently referred to as HSD. Three of the studies reported including individuals with comorbid conditions (i.e., Cornelia de Lange syndrome [45], Emotionally Unstable Personality Disorder (EUPD) [46], and Postural Orthostatic Tachycardia Syndrome (POTS) [47]) while the other seven did not indicate whether patients with mental or physical health comorbidities were excluded from participation.

### Intervention characteristics

Table 2 describes the characteristics and results of the interventions. Nine of the ten studies described multidisciplinary interventions (i.e., interventions which administered more than one treatment modality) to treat EDS/JHS (all except Knowlton et al. [47]). Most of the studies (n = 8) aimed to provide patients with tools and techniques to manage pain independently and overcome functional limitations associated with their conditions, while the other studies (n = 2) focused on minimizing disruptive and harmful behaviours (i.e., self-harm) [45, 46]. CBT was a common psychological treatment used in the included interventions (n = 4) [32, 44, 46, 49]. Half (n = 5) of the studies used a combination of pharmacotherapy (e.g., medications) with some form of psychotherapy (e.g., CBT) [44–46, 51, 52]. The multidisciplinary interventions (n = 9) used a combination of psychological therapy along with physiotherapy, occupational therapy, and/or physical exercise programming. For example, Henry et al. [46] used a combination of Dialectical Behavioural Therapy (DBT), pharmacotherapy, occupational therapy, and physiotherapy.

**Table 2:** Description of Interventions for Individuals with EDS and/or HSD

Study	Single or multi-disciplinary	Goal of the intervention	Description of the psychological intervention	Other intervention components	Intervention administrator	Mode of delivery	Duration and frequency	Intensity	Outcome measures	Results	Intervention modifications/tailoring?
*Cognitive behavioural therapy (CBT)*											
Bathen et al. [32]	Multi	The intervention aimed to provide knowledge and tools for the patient to manage activity limitations and pain in daily life	Cognitive-behavioural (CBT) approach was used for all interventions in the program. Interventions focused on improving pain coping, muscle strength, and muscle endurance. Lectures were delivered by members of a multidisciplinary team; the focus of lectures was on increasing awareness of importance of prioritizing activity and living with pain	Structured exercise program that was easy to perform at home; focused on muscle strength, endurance, core stability, body awareness, physical endurance, and posture. Muscle strength exercises were low resistance with many repetitions	Multidisciplinary team (medical doctor, physiotherapist, registered nurse, social worker, occupational therapist)	In-patient rehabilitation with group program followed by individualized home training	2.5 weeks of hospitalization in a rehabilitation unit followed by 3 months of individualized home training. 25 sessions over 2.5 weeks in hospital	Not clearly described; 25 sessions in total over 2.5 weeks for the in-patient program. No information provided on daily schedule. Each session ranged from 30 to 120 min Home training component consisted of 4–5 exercises 5 days per week	Canadian Occupational Performance Measure (COPM); Tandem walking backwards from UKK test battery of health-related fitness; Stair walking up and down from the Keitel functional test; self-designed test of Stepping up on toes; 13-item Tampa scale (TSK-13); 11-point Numeric Pain Rating Scale (NPRS)	COMP showed significant improvements for activity performance (*P* = 0.008) and performance satisfaction (*P* = 0.005). Physical tests showed significant improvement for Tandem walking backwards (*P* = 0.006), stair walking upwards (*P* = 0.004), and stepping up on toes (*P* = 0.045). Stair walking down showed no significant change. TSK-13 showed significant decrease in kinesiophobia (*P* = 0.022). Six participants had a clinically meaningful (but not significant) decrease in self-reported pain intensity	None
Branson et al. [44]	Multi	The intervention aimed to allow the patient to manage non-acute joint events and pain independently	Two-phase multidisciplinary treatment plan. Phase 1 consisted of psychological pain-management strategies and cognitive behavioural therapy (CBT) techniques learned through outpatient therapy. CBT techniques included breathing exercises, distraction (e.g., engage in enjoyable activities), guided imagery, biofeedback, and behavioural chart (e.g., sticker chart to reward the patient for using pain-management strategies)	Phase 1 included medical stabilization (e.g., removing wires and screws from patient’s jaw). Phase 2 consisted of at-home programs for independent pain management. Pain medication was used at low doses initially, but by the end of phase 1 the patient had creased using all opiates and benzodiazepines. Medications were also used to manage psychological symptoms	Multidisciplinary team (e.g., psychiatrists, psychologists, social workers, pain physicians)	In-patient rehabilitation admissions on an out-patient basis	Phase 1 was 5 months long; Phases 1 and 2 took 21 months in total. Frequency unclear; phase 1 frequency was described as 4 rehabilitation admissions that were 2–3 weeks long each	In phase 1, the patients’ routine included morning attendance at hospital school, afternoon attendance at group therapy, daily physiotherapy, daily appointments with psychologist, and weekly family sessions. In phase 2, the patient was transferred back to local services with weekly reviews at the pediatric ward and referred to a private physiotherapist, and local dentist. The patient began attending regular appointments with a psychologist	Unclear; pain signalling was evaluated	Patient was able to manage her pain independently and competently during acute phases of illness after receiving treatment. She used prescribed medications along with controlled breathing, distraction, and positive self-talk	None
Rahman et al. [49]	Multi	The intervention aimed to manage pain for individuals with Joint Hypermobility Syndrome (JHS), who often respond poorly to analgesics	Cognitive behavioural therapy (CBT) pain management program. Psychologists and physiotherapists worked closely together in sessions so that there was not a distinct boundary between psychology and physiotherapy elements of the program. Both psychologists and physiotherapists applied principles of CBT, although the details of this were not provided	Physiotherapy (note that this also applied CBT principles)	Multidisciplinary team of two clinical psychologists, one nurse, one physiotherapist, and two rheumatologists	In-person out-patient program	6 weeks, 42 h in total; frequency unclear, took place over 8 full days spread out over 6 weeks	Unclear; afternoon and morning sessions (full day)	Pain Self-Efficacy Score (PSEQ), Pain Catastrophising Scale, Depression, Anxiety and Positive Outlook Scale (DAPOS), Brief Pain Inventory (BPI)	Statistically significant improvements were seen in all outcome measures between baseline and 1-month follow-up (*P* ≤ 0.05). Sustained improvements were seen in all outcomes except for average pain intensity at 5-month follow-up, but there was some loss of the improvements measured shortly after the program. Larger percentage improvements were seen for self-efficacy and catastrophizing, while average pain intensity showed the smallest improvements	Each patient sets individual goals that they can work towards, although all sessions take place in a group. Programming was tailored based on the needs of the group (details not provided). Note that this intervention was a pain management program specifically developed for JHS due to previous findings that there was a high attrition rate among JHS patients in heterogenous pain management programs (not fitting in with the group was previously identified as a key reason for dropping out of a pain management program)
Zhou et al. [52]	Multi	The interventions aimed help patients manage their chronic pain associated with Ehlers-Danlos Syndrome (EDS)	Both patients were treated using a multidisciplinary program, daily medications, pain and self-management sessions, cognitive behavioural therapy (CBT), graded exercises, coping, and relaxation strategies Case 1: in addition to above, the patient was provided education around postural awareness and improved body mechanics during work and relaxation Case 2: in addition to above, the patient was also provided with tools for physical and mental relaxation (e.g., mindfulness)	Case 1: Kinesio-taping measures used to stabilize joints. Pain medication Case 2: 6 sessions involved an exercise program	Multidisciplinary team consisting of a pain physician, nurse, pain psychologist, and kinesiologist	In-person; unclear if inpatient or outpatient	6 sessions; frequency and duration not described	6 sessions; intensity not described	Visual Analog Scale (VAS), Pain Disability Interference (PDI)	Case 1**:** Patient expressed significant decrease in pain intensity (VAS) and improvement in her ability to engage in social activity and family responsibilities at 2-month follow-up. Patient continues to be on good pain control and can confidently manage symptoms. P values not provided Case 2: Patient indicated significant improvement in pain symptoms with a decrease in pain intensity (VAS), allowing her to engage in physical activities. P values not provided	None
*Dialectical behavioural therapy (DBT)*											
Henry et al. [46]	Multi	The intervention aimed to improve the patients’ mobility, reduce/stop self-harm, and reduce/stop psychogenic nonepileptic seizures	The Springbank Treatment Programme offers a one-year care pathway of evidence-based treatments for severe personality disorders, including Dialectical Behavioural Therapy (DBT) and a structured programme of activities during the week	Pharmacotherapy, occupational therapy, physiotherapy	DBT therapist; other team members included a physiotherapist and care coordinator	In-patient psychiatric unit	1 year; frequency not described	Not described	Reasons for Living Scale (RFL), Clinical Outcomes in Routine Evaluation (CORE), Generalised Anxiety Disorder 7-point scale (GAD), Difficulties in Emotional Regulation Scale (DERS), The Kentucky Inventory of Mindfulness Skills (KIMS), Personality Assessment Inventory for Borderline personality disorder (PAI-BOR), Patient Health Questionnaire (PHQ-9), Process of Recovery Questionnaire (QPR), and Short Warwick Edinburgh Well-being Scale (SWEMWBS)	Patient improved along all outcome measures at discharge compared to admission scores. This change persisted at the 6 month follow-up. At follow-up, the patient returned to living independently and required no assistance for activities of daily living	None
*Psychoeducation*											
Chaleat-Valayer et al. [48]	Multi	The intervention aimed to allow patients and relatives to understand the disease and treatment, cooperate with caregivers and communicate effectively around the disease, take care of themselves, maintain/increase quality of life, acquire and keep resources to manage their life effectively with the disease	PrEduSED (Programme d’Éducation thérapeutique des patients atteints du SED de type hypermobile), a therapeutic patient education program (TPE). Workshop objectives varied daily from stress management to understanding medications and relaxation	Unclear	Multidisciplinary team (unspecified) including 2 members of French Association of EDS (AFSED). The whole team obtained the whole 40-h training certificate in therapeutic education	In-patient hospitalization	5 days of hospitalization where care alternates with 10 workshops of the program	10 workshops over 5 days; otherwise, unspecified	Coping strategies questionnaire–French version (CSQ-F), Quality of life evaluation (SF-12), Hospital Anxiety and Depression Scale (HAD), Questionnaire d’Image du Corps (QIC), Fatigue Impact Scale (FIS), Hardness scale (Zarit), skills and knowledge quiz, satisfaction questionnaire, vignettes, Goal Attainment Scaling (GAS)	Significant improvement in knowledge/skills (*P* < 0.016 for patients, *P* = 0.016 for relatives); significant improvement in QIC (*P* = 0.047). FIS increased significantly overall and increased for all domains (but only the relationship domain had a significant increase, *P* = 0.05). No significant difference in CSQ-F; no significant impact on HAD or SF-12	Program was initially proposed to be 5 half-days at a day-hospital, but evaluation after the first session led to the program being 5 days of hospitalization where care alternates between 10 workshops
Cravero et al. [45]	Multi	The intervention aimed to minimize disruptive behaviours and intense pain associated with Cornelia de Lange syndrome with comorbid EDS	Psychoeducational treatment that involved a functional analysis of disruptive behaviours, behavioural therapy, and a search for reinforcing factors (e.g., reward and encouragement for desired behaviours)	Medical treatment using nursing and physical care, pain management with level II and III analgesics, and massages. Protective equipment helped reduce bodily injury	Not clearly described; presumably the lead author of the manuscript (psychiatrist)	In-patient hospitalization	3 months; frequency not described	Not described	No concrete measures (physical signs and clinical call points)	Significant clinical improvement was observed after 3 months of hospitalization. Following hospital discharge, patient only needed to visit the emergency room twice to manage brief disruptive behaviours in 2 years. Patient was hospitalized only once in those two years for 3 weeks to handle a shoulder dislocation	None
*Intensive interdisciplinary pain treatment (IIPT)*											
Randall et al. [50]	Multi	The intervention aimed to reduce pain and implement a self-management approach to pain. It was meant to help children restore functioning and remain healthy throughout their lifespan	The Intensive Interdisciplinary Pain Treatment (IIPT) consisted of individual psychotherapy, group-based psychology treatment, family-based treatment, and additional psycho-educational training and support for parents	Individual physical therapy (PT), individual occupational therapy (OT), group-based PT/OT treatment	Multidisciplinary team; not clearly described	In-patient hospitalization	Modal stay was 3 weeks, while mean length of stay was 3.65 weeks (SD = 1.09). Daily frequency	6 h per day: 1 h of individual PT/OT/psychotherapy, 2 h of group-based PT/OT/psychology treatment, 1 h of family-based treatment per day. Parents received 2 h of psycho-educational training and support per week	Functional Disability Inventory (FDI), Numeric Rating Scale (NRS) pain intensity ratings, Pediatric Quality of Life Inventory (Peds-QL)	Majority of respondents reported a significant reduction in pain compared to pre-admission (*P* < 0.001). There was statistically significant decrease in functional disability from admission at 5-years follow-up (*P* < 0.001). There was also clinically significant improvement for pain and function at follow-up. Nearly 80% of respondents characterized themselves as having no functional difficulties at follow-up. 89% graduated from high school on schedule	None; note that eligible patient population was expanded in later years of the program from individuals with Complex Regional Pain Syndrome only
Revivo et al. [51]	Multi	The intervention aimed to teach self-management of pain and stress	The interdisciplinary pain management program was individuated based on the needs of each patient. Common features included psychological counselling, and relaxation training. Psychological interventions included self-management of pain and stress (e.g. relaxation techniques) and coping self-statements during distress	Physical therapy, occupational therapy, weekly pediatric rehabilitation medicine follow-up, medication management (e.g., correcting poor sleep)	Multidisciplinary team including a pediatric physiatrist, psychologist, therapist, and physician. Psychological interventions implied to be administered by a psychologist	In-person administration at an out-patient clinic	4–8 weeks; 1–2 sessions per week	1–2 half-day sessions per week, lasting about 3–4 h per session	Numeric Rating Scale, Bath Adolescent Pain questionnaire (BAPQ), Parent Bath Adolescent Pain-Parental Impact Questionnaire (BAP-PIQ)	Average pain intensity ratings and multiple domains of patient/parent functioning improved significantly from pre-treatment to post-treatment (*P* < 0.05). There were also statistically significant improvements in daily functioning (social and physical) (*P* < 0.05). 97% returned to school and most patients returned to valued activities (e.g., music, sports, theater, crafting). There was significant reduction in depression, general and pain-related anxiety (*P* < 0.05). Unexpectedly, Joint Hypermobility Syndrome (JHS) patients reported a decline in family functioning	The pain management program was individuated based on patient need, although treatment plans had common features
*Acceptance and commitment therapy (ACT)*											
Knowlton et al. [47]	Single	The intervention aimed to help the patient develop psychological flexibility when faced with thoughts, feelings, and behaviours associated with pain, ultimately improving quality of life overall	More to Life, a standard Acceptance and Commitment Therapy (ACT) protocol, was implemented. Sessions focused on techniques to foster psychological flexibility related to chronic pain and resulting depression. Patient engaged in a creative hopelessness exercise, exploration of solutions to live with her chronic medical condition, and mindfulness techniques	N/A	Post-master’s clinical psychology PhD student provided treatment; licensed clinical psychologist experienced in ACT supervised	In-person administration at an outpatient clinic (psychology department-based integrated care training clinic)	8 months; frequency unclear	18 therapy sessions lasting 45–50 min each over the course of 8 months	Patient-Reported Outcomes Measurement Information System (PROMIS-29), Acceptance and Action Questionnaire (AAQ-II), Psychological Inflexibility in Pain Scale (PIPS), Reliable Change Index (RCI), Wechsler Abbreviated Intelligence Scale (WASI-II)	There were significant improvements in depression, psychological inflexibility, and relationship between psychological inflexibility and pain. Patient made significant improvements in functioning, but fatigue had profound negative impact on session engagement. *P* < 0.05 for all significant results	None

JHS would be presently known as HSD

Interventions were administered by a multidisciplinary team for most of the studies (n = 8) [32, 44, 46, 48–52], while one study did not clearly describe the administrators [45] and one study had only one administrator (post-master’s clinical psychology Ph.D. student) [47]. For all ten studies, the administrator of the psychological intervention specifically was not clearly described, although it could be assumed that the psychologists and therapists were the administrators for those interventions which listed these professionals as part of the team. All interventions were administered in person; six interventions were administered on an in-patient basis (in clinic or in hospital) [32, 44–46, 48, 50], three interventions were administered on an out-patient basis [47, 49, 51], and one study did not describe whether the intervention was delivered on an in-patient or out-patient basis [52]. The duration of the interventions ranged from 5 days to 21 months for nine studies, while one study did not clearly describe the length of the intervention [52]. The intensity of the interventions was not clearly described for seven studies [32, 44–46, 48, 49, 52]; the intensity of the other three interventions ranged from 45 min to 6 h in length per day [47, 50, 51].

In terms of outcome measures, two of the studies did not outline clear outcome measures [44, 45], while the other eight listed multiple outcome measures which included at least one that measured pain levels. All the interventions resulted in some improvements in outcome measures from admission to follow-up (i.e., decrease in pain, decrease in depression), although two studies did not report significant results [44, 46]. Three of the interventions received modifications and/or tailoring before or during their administration: Chaleat-Valayer et al. [48] altered the program to be 5 days of hospitalization instead of 5 half-days at a day hospital (reasoning unclear; the decision was made following initial evaluation of the sessions), Revivo et al. [51] individuated treatment plans based on patient need (details not provided), and Rahman, Daniel, and Grahame [49] tailored the daily program design to accommodate the needs of each group of patients receiving treatment over the years (details not provided).

### Psychological treatment component characteristics

Four of the studies included treatments using CBT techniques as the psychological intervention, one study investigated a DBT approach, two of the studies investigated programs focused on psychoeducation, two of the studies applied an intensive interdisciplinary pain program (IIPT), and one study used an ACT approach.

#### Cognitive behavioural therapy (CBT)

CBT is a form of psychotherapy that is commonly used to address pain management, disability, and other mental health challenges in individuals with chronic illness by providing the patient with tools to identify and cope with these challenges [32]. Bathen et al.’s cohort study [32] investigated a program that used a CBT approach for all intervention components (e.g., exercise groups, pain management groups, and lectures) which were aimed at providing participants with EDS hypermobility type (EDS-HT) and JHS with tools to manage pain and activity limitations. Additional details about the approach were not provided. The rehabilitation program took place over the course of 2.5 weeks and session lengths ranged from 30 to 120 min. It was followed by a 12-week at-home exercise program. The program resulted in a significant change in the perceived performance of daily activities, a significant increase in muscle strength and endurance, and a significant reduction in kinesiophobia.

Branson et al.’s case report [44] investigated a 21-month two-phase program consisting of regular psychologist appointments along with other treatment sessions (e.g., physiotherapy and medication) to manage pain and non-acute joint events in patients with EDS-HT and JHS. The participant was taught pain management and CBT methods (i.e., breathing techniques, positive self-talk, and distraction). Session lengths and intensity were not clearly described. After the first five months of inpatient treatment, the participant continued to receive outpatient psychological treatment. The participant was ultimately able to manage her pain competently using CBT techniques in conjunction with pain medications (mirtazapine, gabapentin, and acetaminophen and naproxen, as needed).

Rahman, Daniel, and Grahame’s cohort study [49] investigated a 6-week multidisciplinary pain management program that used CBT techniques for individuals with JHS-related pain. The program consisted of psychology sessions as well as physiotherapy and multidisciplinary sessions over 8 full days (42 h in total). Psychologists and physiologists both used CBT principles and worked closely with each other in sessions, although the principles were not specified in the study. Ultimately, participants had statistically significant improvements in all outcome measures (i.e., self-efficacy, depression, anxiety, frustration, impact of pain, average pain intensity, and pain catastrophizing) between baseline and 1-month follow-up, most of which were sustained at 5-month follow-up.

Zhou, Rewari, and Shanthanna’s two case reports [52] investigated a 6-session pain management program for individuals with EDS. Both patients were treated using a multidisciplinary program, daily medications, pain and self-management sessions, CBT, graded exercises, coping, and relaxation strategies by a multidisciplinary team of pain physicians, nurses, pain psychologists, and kinesiologists. In addition to the above, the patient in case report 1 was provided education about postural awareness and improved body mechanics during work and relaxation, and the patient in Case 2 was also provided with tools for physical and mental relaxation (e.g., mindfulness). Session intensity and lengths were not described. Ultimately, both patients had significantly improved pain control and improvement in daily living post-intervention.

#### Dialectical behavioral therapy (DBT)

DBT is a type of CBT that uses strategies such as mindfulness, distress tolerance, and emotion regulation to improve the patient’s quality of life [46]. Henry et al.’s case report [46] investigated a year-long inpatient treatment program *(Springbank Treatment Programme)* aimed at reducing self-harm and promoting independence in a patient with EUPD, as well as comorbid EDS (unspecified type) and Functional Neurological Disorder. The program consisted of DBT as the psychological treatment along with pharmacotherapy, occupational therapy, and physiotherapy. The DBT portion of the treatment included emotional regulation and mindfulness techniques to reduce self-harm tendencies. The participant is noted to have completed two cycles of DBT skills training along with 12 months of one-on-one sessions, but intensity and frequency were not described further than that. The participant saw improvements in all nine outcome measures assessing symptomology, quality of life, mindfulness, and recovery at discharge which persisted at 6-month follow-up. It is notable that the DBT was used primarily to reduce self-harm behaviours associated with the participant’s comorbid severe EUPD, while her EDS-related mobility problems were primarily targeted by physiotherapy.

#### Psychoeducational programs

Psychoeducation, also known as therapeutic patient education, helps patients with chronic diseases manage their lives effectively through education about their disease and treatment [48]. Chaleat-Valayer et al.’s cohort study [48] investigated a therapeutic education program *(PrEduSED)* for patients with hypermobile EDS that consisted of 10 workshops. Each workshop was structured around a unique objective (e.g., to teach relaxation, to educate about EDS) and the program took place over 5 days. The program was designed for both the patients with EDS as well as their relatives. Overall, patients saw a significant improvement in knowledge/skills, body image, and relationships but no significant changes were seen in terms of quality of life or pain coping strategies. Relatives had a significant improvement in skills and knowledge at the 6-month follow-up.

Cravero et al.’s case report [45] featured a psychoeducational treatment aimed at correcting challenging and disruptive behaviours causing bodily injury in patients with Cornelia de Lange Syndrome with comorbid EDS (classic type). This was administered in conjunction with medications to manage symptoms. This program took place over the course of 3 months during which the patient was hospitalized. The exact duration and intensity of the program were unspecified. The psychoeducational component of treatment involved a functional analysis of disruptive behaviours (i.e., identifying variables that influence challenging behaviours) and reinforcement of desired behaviours. Overall, significant clinical improvements were seen after 3 months of hospitalization; in the 2 years following discharge, the patient had a reduction in emergency room visits.

#### Intensive interdisciplinary treatment programs (IIPT)

IIPT treats chronic pain using a biopsychosocial model of health and illness and involves coordinated psychological and physical care. It may include nutrition, recreation, and expressive art therapies in addition to physical/occupational/CBT therapies [50]. Randall et al.’s cohort study [50] investigated an IIPT ranging from 3 to 6 weeks of daily therapy for patients with EDS and other pain disorders (e.g., Complex Regional Pain Syndrome). The psychological aspect consisted of psychotherapy (individual and group) for the patient (ages 8–19) and psychoeducational training for parents (not described). Other aspects of the program included individual/group physical therapy and occupational therapy. Details about the sessions were not provided beyond this description. Patient sessions (all aspects of the program) occurred over the course of 6 h daily while educational sessions for parents occurred over the course of 2 h per week. All outcome measures were measured at 5 years post-treatment. Ultimately, there was a statistically significant decrease in functional disability and clinically significant improvement was found for pain and function. Nearly 80% of respondents reported having no functional difficulties at follow-up, and 89% graduated high school on schedule.

Revivo et al.’s cohort study [51] also investigated an IIPT lasting 4–8 weeks that aimed to help patients with hypermobile type EDS and JHS with pain management. The program consisted of psychological counselling along with physical therapy, occupational therapy, and medication management. The psychological counselling included discussions of self-management strategies for pain and stress (e.g., coping self-statements) and relaxation techniques. Participants had 1–2 sessions per week lasting approximately 3–4 h each. Overall, participants saw a statistically significant reduction in average pain intensity ratings, improvements in physical and social daily functioning, and reductions in depression and pain-related anxiety. Almost all (97%) participants returned to school and most patients returned to valued activities (i.e., music, sports, etc.).

#### Acceptance and commitment therapy (ACT)

ACT is used to treat chronic pain and is a promising alternative for those who do not respond to CBT. It focuses on psychological flexibility and emphasizes accepting what’s out of one’s control and engaging in actions that will allow for a fulfilling life [47]. Knowlton et al.’s case study [47] investigated an 8-month program called *More to Life*, which utilized an ACT approach for a patient with vascular type EDS. The program aimed to encourage patients to engage in actions that allow for a fulfilling life in face of disability by increasing psychological flexibility. This study was the only one to describe and evaluate a single discipline intervention. In this case study, the participant was taught ACT techniques over the course of 18 therapy sessions, lasting 45–50 min each. Each session focused on a different area of mindfulness and psychological flexibility (e.g., acceptance, core skills exercise, etc.). Ultimately, the participant saw clinically significant improvements in depression, psychological inflexibility, and flexibility related to pain.

## Discussion

The purpose of this scoping review was to identify the nature and extent of studies investigating psychological interventions for individuals with EDS and HSD (formerly known as JHS). This review included five cohort studies, four case reports, and one study with two case reports. All included studies evaluated psychological interventions for patients with EDS and/or HSD. The most reported intervention was CBT. Notably, majority of participants were female across all studies. This is aligned with the prevalence of EDS and HSD in males and females; a UK cohort study showed that out of 6021 individuals diagnosed with EDS or HSD, roughly 70% were women [53]. We also found that the majority of the treatment paradigms for EDS and/or HSD were multidisciplinary. It should be noted that the psychological components of the treatment plans were poorly described; many of the studies did not provide information detailing the intensity, duration, and/or frequency of interventions. Additionally, the results of case studies may not be generalizable to others within the cohort, and many of the cohort studies had relatively small sample sizes (n < 50). Overall, our findings show that there is a lack of high-quality research on psychological interventions for individuals with EDS and HSD. This is especially problematic because of the high prevalence of psychiatric disorders among individuals with EDS and HSD, which is becoming increasingly evident [54, 55]. Our review adds to the existing commentary about the significance of psychological burden for these individuals while emphasizing the alarming lack of research in this area despite a critical need for appropriate treatments.

### Notable psychological interventions within included studies

Most included studies reported significant improvements for their respective interventions in terms of pain, reduction of destructive behaviours, and/or other outcome measures including anxiety, depression, and quality of life. However, these results are inconclusive due to the lack of high-quality research surrounding psychological interventions for individuals with EDS and HSD (e.g., small sample sizes, study types with poor validity/reliability, lack of RCTs, etc.). Some interventions identified in our review have been previously explored in similar patient populations and are thus recommended for further investigation in patients with EDS and/or HSD.

Four of our included studies investigated a CBT approach. Notably, Bathen et al.’s cohort study [32] reported significant improvements in activity performance, performance satisfaction, physical strength and endurance, self-reported pain intensity, and a decrease in kinesiophobia. Rahman, Daniel, and Grahame’s cohort study [49] reported significant improvements in pain and depressive symptoms. In accordance with these results, several meta-analyses and reviews have demonstrated the efficacy of CBT for managing pain and improving daily functioning and independence in patients with chronic pain disorders [56, 57]. For example, a review by Knoerl et al. [58] investigated CBT for adults with chronic pain associated with fibromyalgia and arthritis. The CBT treatment reduced pain intensity significantly in 43% of the included RCTs, and significant improvements in other outcomes such as depression and anxiety were also noted in over 50% of the trials testing these outcomes. However, it should be noted that many trials on CBT for individuals with chronic pain have not compared CBT groups to another active control group. Williams et al.’s systematic review [59] demonstrated that when compared with active controls, some small improvements were observed for CBT in terms of pain, disability, and distress for adults with chronic pain, but these improvements were not significant. While we cannot conclude the efficacy of CBT based on our study results due to a lack of high-quality research, CBT may be further investigated as a method of improving pain and daily functioning of patients with conditions such as EDS or HSD which also cause chronic pain and disability.

Two studies investigated a psychoeducational program. Notably, Chaleat-Valayer et al.’s cohort study [48] found significant improvements in the patients’ self-image as well as the knowledge and skills of patients and their relatives. A 2021 systematic review by Gómez-de-Regil [60] on psychoeducational interventions for patients with fibromyalgia revealed that interventions were able to significantly improve patients’ pain intensity, fatigue, sleep quality, depression, anxiety, functional ability cognitive impairment, and quality of life. These results are supported by studies investigating psychoeducation for individuals with chronic pain [61, 62]. While we cannot conclude the efficacy of psychoeducational programs for those with EDS/HSD due to a lack of high-quality research, research on similar patient populations shows that psychoeducational programs may be worth investigating to improve the knowledge and skills of individuals who live with chronic pain and disability (e.g., patients with EDS and their families).

Two studies investigated an IIPT program. Randall et al.’s cohort study [50] found significant decreases in functional disability from admission to follow-up, as well as significant improvements in pain and function. Revivo et al.’s cohort study [51] found significant improvements in average pain intensity, patient/parent functioning, daily functioning, depression, and pain-related anxiety. These findings are consistent with results from a systematic review investigating IIPTs for children with chronic pain [63], which found IIPTs may significantly improve pain symptoms as well as disability short-term; however, the review revealed that there is a lack of well-designed clinical trials investigating IIPT efficacy, so these conclusions may not be reliable. An observational study published by Zernikow et al. [64] investigated IIPTs for adolescents and children with chronic pain. The authors found that participants had a significant reduction in pain-related and psychological (anxiety, depression, pain catastrophizing) outcome measures, which were sustained at the four-year follow-up. Overall, previous studies investigating IIPTs for the management of pain and other symptoms have yielded promising results for the chronic pain population. Although we again cannot conclude the efficacy of IIPT for those with EDS/HSD, this intervention warrants further investigation with high-quality studies for this population.

### Potential psychological interventions not explored in our included studies

It is notable that some psychological interventions studied in chronic pain populations, which have demonstrated significant improvements in outcomes such as pain, depression, and anxiety, have not been studied in individuals with EDS and/or HSD, or at least were not identified in this review. For example, Motivational Interviewing (MI) is a technique focusing on resolving an individual’s ambivalence toward behavioural change by strengthening their commitment and motivation to change [65]. MI has been previously investigated for individuals struggling with chronic pain; a systematic review and meta-analysis by Alperstein and Sharpe [65] on MI for individuals with chronic pain identified that MI was able to significantly increase short-term adherence to prescribed treatment plans and significantly reduce pain in the short-term. While more research is required to validate these results, future studies may explore MI as a psychological approach for improved pain management and adherence to treatment plans for individuals with EDS and HSD.

Mindfulness-Based Stress Reduction (MBSR) is another promising psychological intervention for individuals with EDS/HSD. MBSR promotes a non-judgemental approach to pain where the physical and psychological aspects of pain are uncoupled [66]. This is typically achieved through meditation and mindfulness practices in which the patient increases their awareness of body signals and breath. Some of the studies in our review briefly outlined mindfulness techniques (e.g., relaxation), but none of the programs clearly identified or described mindfulness as the main psychological component. MBSR has been previously investigated as a treatment for pain in individuals with chronic lower back pain, headache, and fibromyalgia [66]. A 2011 systematic review [67] found that MBSR may significantly decrease anxiety, stress, and depression while improving the quality of life of patients with diseases such as cancer as well as chronic pain disorders. Another systematic review by Abbott et al. [68] found similar results for patients with vascular disease in that MBSR was effective in improving depression, anxiety, and psychosocial stress. Thus, future studies should explore MBSR in treating psychological symptoms associated with EDS and HSD as well.

### Notable gaps in existing research and future direction

Our review yielded only ten studies that investigated psychological interventions for individuals with EDS and/or HSD. Of these ten, half were single case reports; the remaining were retrospective cohort studies. Consequently, study results may not be generalizable to the overall cohort. RCTs should be conducted to reliably evaluate the effectiveness of these psychological interventions. Qualitative studies were also not identified in our review but may be a meaningful addition to the literature (i.e., to provide insight into patient experience). While it is encouraging that the existing research has focused on individually tailored programs given that research has shown the importance of tailored treatment plans in the successful management of chronic pain, future studies should investigate the efficacy of tailored treatment plans on a larger cohort for increased validity and reliability [69]. Similarly, small sample sizes may affect the reliability of the cohort study outcomes. Two of the studies [32, 51] explicitly recommended future studies use larger sample sizes to validate the results. Another notable limitation among included studies was the lack of validated outcome measures used in some studies. Branson et al.’s case report [44] relied on the patient’s self-report and physical symptoms, while Cravero et al.’s case report [45] relied on physical signs. Future studies should use standardized outcome measures for increased validity of findings. Lastly, one case report [46] included in our study focused heavily on the patient’s other comorbidity (EUPD) rather than their EDS, which may confound its findings (i.e., Henry et al.’s [46] intervention may be better suited for individuals with EUPD rather than EDS).

An important finding among our included studies is that the psychological interventions were often not well-described. Many of the interventions mentioned the underlying psychological methods in general terms (e.g., CBT techniques), but failed to describe the treatment details. It was thus often unclear where and how the technique was applied. For instance, Rahman, Daniel, and Grahame [49] stated that the administrators in their study applied principles of CBT in their interventions, but the authors did not provide further details regarding which principles were used and how those principles were integrated into the treatment plan. Furthermore, in many of the studies, the administrators of the psychological interventions were not clearly identified (e.g., described as a “multidisciplinary team”) and it was also unclear what type of training these administrators received to provide these interventions. Future studies should outline the psychological interventions in greater detail in terms of their principles, how those principles were applied to the treatment plan, and who the treatment administrators were. Similarly, many studies did not provide details on participant demographics. The race/ethnicity of participants was provided for only four of the ten studies, and all participants were white/Caucasian. Future studies should include a more detailed demographic profile of its participants to establish the external validity of the treatments for a diverse population (e.g., determine if an intervention is suitable for individuals of different ethnicities).

### Strengths and limitations

To our knowledge, this is the only existing scoping review that examines the literature on psychological interventions for individuals with EDS/HSD and identifies various gaps in existing research. It is strengthened by using an experienced information specialist to conduct an exhaustive literature search, as well as multiple reviewers to conduct screening and data extraction independently, in duplicate. The multidisciplinary expertise of our research team (e.g., EDS content experts, methodology experts) helped to strengthen the review, as well as our lifespan approach (e.g., we broadened our search and inclusion criteria to investigate individuals of all ages, rather than just adult or pediatric patients). We also acknowledge some limitations. Since this review included English-language studies only, there may have been a bias toward the inclusion of studies from English-speaking countries. Additionally, we limited our grey literature to three websites (Ehlers-Danlos Society, Ehlers-Danlos Support UK, and Marfan Foundation) on the recommendation of the research team; consequently, we may have excluded important and relevant studies from other sources. Another possible limitation is the search of only MEDLINE, CINAHL, EMBASE, and PsycINFO databases; a wider array of databases may have yielded more articles for inclusion. Additionally, our literature search was conducted on March 28, 2021; thus, we were only able to include articles that had been published by this date, all of which happened to investigate in-person psychological interventions. We acknowledge that we may have missed interventions that were subsequently conducted and published, such as the online RCT by Kalisch and colleagues [70]. It should also be noted that nine of the ten studies were multidisciplinary, meaning that the psychological intervention was only one part of the treatment plan. As a result, it is impossible to ascertain that the outcomes of the treatments were a result of the psychological interventions alone, meaning that the included studies in our review may not accurately reflect the effectiveness of psychological interventions (e.g., the outcomes may have been more influenced by other components of the treatment such as medication or physiotherapy). Overall, more studies are needed that focus on individuals with EDS/HSD to verify the effectiveness of such interventions.

### Conclusion

This scoping review on psychological interventions for individuals with EDS and/or HSD identified a lack of high-quality research for this population. Only cohort studies and case reports were identified, and most of the participants were diagnosed with EDS hypermobility type or JHS/HSD. CBT, IIPT, and psychoeducation may be worth investigating further with high-quality studies, but no conclusions about their efficacy can be made presently. MI and MBSR are psychological interventions that may be effective in treating pain and symptoms associated with pain (e.g., depression, anxiety). These may be further investigated for individuals with EDS and HSD. Future studies should also investigate psychological interventions with participants with other forms of EDS, as well as with larger sample sizes and higher quality studies (e.g., RCTs). Furthermore, many of the studies included in our review lacked sufficient detail surrounding demographic information (i.e., race/ethnicity) and description of the intervention (i.e., how the principles of CBT were applied to the treatment plan). This information should be provided in future studies to account for demographic variables in determining the efficacy of a treatment plan and to provide a clearer understanding of effective psychological techniques. Ultimately, many patients with EDS and HSD experience heightened psychological distress from their conditions, yet there is a lack of high-quality research investigating psychological interventions for these individuals, both alone and as a component of a multidisciplinary treatment plan. The results of our study may guide future researchers in designing high-quality studies to determine the most effective interventions for the rehabilitation of individuals with EDS and HSD, which may later be useful in guiding clinical practice.

### Supplementary Information

**Additional file 1:** Search strategy from CINAHL.

## Data Availability

The data used and/or analyzed in the current study are available from the corresponding author upon reasonable request.

## Abbreviations

EDS: Ehlers-Danlos syndrome
JHS: Joint hypermobility syndrome
HSD: Hypermobility spectrum disorder
CBT: Cognitive behavioural therapy
iKT: Integrated knowledge translation
ACT: Acceptance and commitment therapy
RCT: Randomized controlled trial
TIDieR: Template for Intervention Description and Replication
EUPD: Emotionally unstable personality disorder
POTS: Postural orthostatic tachycardia syndrome
DBT: Dialectical behavioural therapy
IIPT: Intensive interdisciplinary pain treatment
EDS-HT: EDS hypermobility type
MI: Motivational interviewing
MBSR: Mindfulness-based stress reduction
