# Supplementary material for: Psychological interventions for individuals with Ehlers-Danlos syndrome and hypermobility spectrum disorder: a scoping review
Source: Orphanet J Rare Dis. 2023 Aug 31;18:254. doi: 10.1186/s13023-023-02799-y (PMC10472575; doi:10.1186/s13023-023-02799-y)
Supplement: Supplementary file 1 — Additional file 1: Search strategy from CINAHL. [file 13023_2023_2799_MOESM1_ESM.docx]

**Additional file 1**

(MH "Ehlers-Danlos Syndrome")

OR

(TX ehlers-danlos) OR (TX "ehlers danlos") OR (TX "cutis elastic") OR (TX "joint mobility") OR (TX JHS) OR (TX BJHS) OR (TX hypermobil*) OR (TX hyper-mobil*)

AND

(MH "Autogenic Training (Iowa NIC)") OR (MH "Behavior Therapy+") OR (MH "Counseling+") OR (MH "Family Therapy") OR (MH "Health Education") OR (MH "Hypnosis") OR (MH "Internet-Based Intervention") OR (MH "Mental Health") OR (MH "Mind Body Techniques") OR (MH "Psychotherapy+") OR (MH "Problem Solving") OR (MH "Psychopathology") OR (MH "Psychotherapy, Psychodynamic") OR (MH "Relaxation Techniques+") OR (MH "Stress, Psychological") OR (MH "Telephone+") OR (MH "Therapy, Computer Assisted")

OR

(TX autogenic*) OR (TX anxiety) OR (TX "acceptance therap*") OR (TX behavior*) OR (TX behaviour*) OR (TX biofeedback) OR (TX "cognitive restructur*") OR (TX "commitment therap*") OR (TX counselling) OR (TX counseling) OR (TX cognitive N4 therap*) OR (TX cognitive N4 intervention*) OR (TX cognitive N4 treatment*) OR (TX CBT) OR (TX "computer assist*") OR (TX "cell* phone*") OR (TX distress*) OR (TX "exposure therap*) OR (TX "emotion focused therap*") OR (TX family N4 intervention*) OR (TX family N4 treatment*) OR (TX family N4 therap*) OR (TX goal* N4 setting) OR (TX "guided image*") OR (TX health N4 educat*) OR (TX hypnotherap*) OR (TX hypnosis) OR (TX interoception) OR (TX interoceptive) OR (TX "lifestyle modifi*") OR (TX "interpersonal therap*") OR (TX internet) OR (TX "mind body therap*")

OR

(TX "mind-body therap*") OR (TX motivat* N4 interv*) OR (TX motivat* N4 enhanc*) OR (TX "mental health") OR (TX MBCT) OR (TX MBSR) OR (TX manag* N4 anxiety) OR (TX manag* N4 depress*) OR (TX meditat*) OR (TX mindfulness) OR (TX "mobile phone*") OR (TX online) OR (TX psychopathol*) OR (TX psychosocial*) OR (TX psycho-educat*) OR (TX psychoeducat*) OR (TX psycholog* N4 therap*) OR (TX psycholog* N4 treatment*) OR (TX psycholog* N4 intervention*) OR (TX psychotherap*) OR (TX psycho-therap*) OR (TX "problem solving") OR (TX psychodynamic N4 therap*) OR (TX psycho-dynamic N4 therap*) OR (TX relax*.mp) OR (TX "self manage*") OR (TX "skill* train*") OR (TX stress N4 manag*) OR (TX smartphone*) OR (TX teletherap*) OR (TX telephone*) OR (TX "web based")
